# Bioprospecting White-Rot Basidiomycete *Irpex lacteus* for Improved Extraction of Lignocellulose-Degrading Enzymes and Their Further Application

**DOI:** 10.3390/jof6040256

**Published:** 2020-10-29

**Authors:** Linda Mezule, Anna Civzele

**Affiliations:** Water Research and Environmental Biotechnology Laboratory, Riga Technical University, P. Valdena 1-303 Riga, Latvia; anna.civzele@rtu.lv

**Keywords:** lignocellulosic biomass, enzymatic hydrolysis, enzymes, white rot fungi

## Abstract

Lignocellulosic biomass can be used as a source for energy, fuel and valuable chemical production. From all available technologies, biological approaches have been recognized as the most environmentally friendly and sustainable ones. At the same time, high conversion costs, low efficiency and environmental issues still hinder the introduction of biological processes into industrial scale manufacturing. The aim of this study was to determine the most suitable enzyme cocktail recovery conditions from a biomass–fungal culture of the white-rot basidiomycete *Irpex lacteus*. Subsequent evaluation of the overall enzyme cocktail efficiency to release fermentable carbohydrates from biomass showed that prolonged fungal cultivation decreases the quality of the produced enzyme cocktail. At the same time, introduction of ultrasound pre-treatment during enzyme extraction improved the recovered enzyme cocktail efficiency in converting biomass to fermentable sugars, yielding up to 0.25 g of fermentable sugar per g dry hay biomass and up to 0.11 g per g dried straw or microalgae substrates. The results demonstrated that the production of lignocellulose-degrading enzymes from fungi is more sensitive than previously described, especially in terms of fungal growth, culture sterility and incubation conditions.

## 1. Introduction

According to the European Directive 2018/2001, by 2030 a total of 32% of energy consumption should be obtained from renewable sources; moreover, land should not be converted to accommodate the production of agricultural raw material for biofuels, bioliquids and biomass fuels [1]. In 2018, renewable energy accounted for 21% of the total energy used (and in 12 countries more than 32%) for heating and cooling in the European Union (EU) [2]. At the same time, introduction of renewable energy sources into the transport sector is still the most challenging area. In 2018, only two EU countries were above the 2020 target share of 10%: Sweden (29.7%) and Finland (14.4%) [3]. The most widely used renewable energy sources in transport are liquid biofuels (bioethanol, biodiesel), hydrogen and methane. One of the resources for fuel production that is facilitated by various regulations, the scientific community and society in general is lignocellulosic biomass, e.g., agricultural waste, wood processing waste, straw and other materials unusable for human consumption. Despite the high potential of the biomass material, challenges related to efficient conversion still remain open and limit its extensive use [4]. To convert lignocellulosic biomass into biofuels, four major treatment steps (pre-treatment, hydrolysis, fermentation and product recovery) must be performed [5]. These, when compared to production of fuel from fossil resources, biomass burning or agricultural crops, are still uncompetitive in terms of price and efficiency; however, their environmental importance is recognized worldwide.

In biomass hydrolysis, the most commonly used methods are chemical (acid, alkali) or biological (enzymes). A common trend is to combine these approaches with various pre-treatment techniques to improve the overall performance and accessibility of the enzymes (if biological hydrolysis is used) or to minimize the recalcitrance of the substrate as such. From all technologies, biological approaches have been recognized as the most sustainable ones, due to their non-hazardous waste, biodegradability and recirculation potential. However, the need for specific and expensive lignocellulose-degrading enzymes, the impact of certain commercial enzyme manufacturers and the generally slow conversion rates (from one to several days) still limit the use of this technology in industrial scale production [6].

The industrial pursuit of obtaining a high level of fermentable sugars from lignocellulosic biomass depends substantially on the successful expression and blend of a wide range of enzymes in one complex. However, the challenge to successfully assemble an entire suite of these enzymes that can function optimally at the same time and under different conditions to completely digest lignocellulosic biomass to simple sugars still remains [7]. Furthermore, the evaluation of the most appropriate microbial species for production of natural enzyme mixes is still ongoing [8,9].

Improved enzyme productivity has been related to many factors, e.g., use of various fungal and bacterial species [7,8], optimization of growth conditions [10,11], substrate type and biomass pre-treatment prior to inoculation [12,13]. At the same time, combined assessment of enzyme production with extraction efficiency has not been thoroughly evaluated, resulting in potential protein losses during the extraction process. The aim of this study was to determine the most suitable enzyme cocktail recovery conditions from biomass-fungal cultures to generate more effective natural enzyme products. *Irpex lacteus* was used as the representative species for the lignocellulose-degrading enzyme production due to its prior recognizability [8,14], ability to produce full array of enzymes for lignin, cellulose and hemicellulose degradation [15] and high colonization potential on various wood types [16]. Thus, the efficiency of the produced enzymes was not demonstrated as an activity of a single CAZyme, e.g., cellulase, xylanase, but as the overall enzyme cocktail efficiency to convert biomass into fermentable sugars.

## 2. Materials and Methods

### 2.1. Fungus and Culture Conditions

Carbohydrate-degrading enzymes were produced from white-rot basidiomycetes *Irpex lacteus* (Fr.) Fr. IBB 104 that were obtained from Durmishidze Institute of Biochemistry and Biotechnology, Georgia. Stock cultures were maintained on potato dextrose agar (4 g/L potato extract, 20 g/L glucose, 15 g/L agar, Oxoid Ltd., Basingstoke, Hants, UK) at 2–8 °C.

### 2.2. Lignocellulosic Substrates

**Figure 1:**
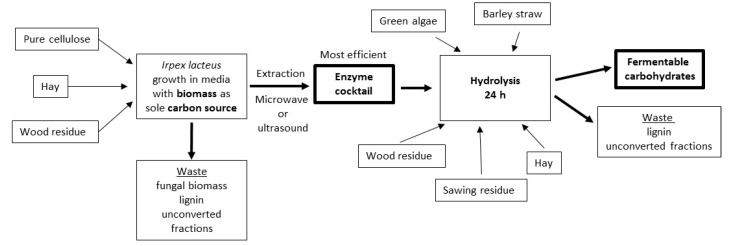
Experimental flow scheme showing the biomass materials used for enzyme production and biomass used for the hydrolysis tests of the most efficient enzyme product obtained after the extraction process adjustment (Section 2.4.). Biomass was not mixed during the experiments.

Within the study a single batch of hay, wood residue chips and cellulose (Avicel PH-101, Sigma-Aldrich, Darmstadt, Germany) substrates were used for enzyme production. To evaluate the yields and efficiency of hydrolysis with the produced enzymes, hay, wood residue chips, sawing residue chips, barley straw and green microalgae *Desmodesmus communis* CCAP 276/4B were used as substrates (see Figure 1 for process flow).

Hay (dry weight (DW): 92.8 ± 1.3%; ash 6.03%) was collected from semi-natural grassland in Latvia in 2019. Wood residue chips (DW: 93.4%) were obtained from a forest logging company and consisted mainly of aspen and spruce waste. Sawing residue chips were obtained from a carpentry company in Latvia. In total, 95% of the material originated from birch, pine and spruce. Barley straw (DW: 94.2%) was harvested in Latvia in 2019. In all tests a single batch of biomass was used to avoid compositional variation between the repetitions. Before use, hay, straw, wood and sawing chips were milled by a mechanical cutting mill (Retsch SM100, Haan, Germany) with 1.5 kW drive and a parallel section rotor with a peripheral speed of 9.4–11.4 m/s to obtain fractions <0.5 cm. Microalgae were cultivated in liquid modified Bold’s Basal Medium with 3-fold nitrogen and vitamins [17] under artificial sunlight (white fluorescent light with the photosynthetically active radiation of 100 µmol m^2^/s and 16:8-h lighting regime) in screw cap bottles with tube connection holes and constant pre-filtered air supply (8 mL/h) that was enriched with 1% CO_2_. Algal biomass harvesting was performed by centrifugation (8500 RCF, 10 min).

### 2.3. Enzyme Production

Prior to enzyme production, pieces of stock fungal culture were incubated in 300 mL Erlenmeyer flasks containing 0.8 g KH_2_PO_4_, 0.4 g K_2_HPO_4_, 0.5 g MgSO_4_·7H_2_O, 2 g NH_4_NO_3_, 2 g yeast extract and 10 g glucose per L (pH 5.3–5.5) for 3–4 days in an orbital shaker (150 rpm, 30 °C, New Brunswick™ Innova^®^ 43, Eppendorf Austria GmbH, Wien, Austria). The grown liquid culture was homogenized (1 min, 400 rpm, Retsch GM 200, Haan, Germany) and then used for inoculation in the previously described liquid broth where glucose was replaced with 4% of hay, wood chips or Avicel (Figure 1). All flasks were incubated in an orbital shaker at 150 rpm for 4 days at 30 °C. Higher solids content was avoided to ensure good mixing.

### 2.4. Enzyme Extraction

After incubation, the liquid fraction was either centrifuged for 10 min at 8500 RCF to remove smaller solids or prior to centrifugation was treated with ultrasound (ultrasonication at 30 or 50 Hz for 2 or 4 min) or microwave (5 min, 1250 W). The supernatant was then collected by centrifugation (8500 RCF for 10 min.), and proteins were precipitated with 50% *w*/*v* ammonium sulfate and stored at 4 °C for 24–48 h. The obtained sediments were extracted with centrifugation for 10 min at 8500 RCF. After centrifugation, equal volumes of 0.05 M sodium citrate buffer were added to each extract and the products were stored at 4 °C.

Additionally, biomass treatment with ultrasonication (30 or 50 Hz for 2 or 4 min) and microwave (5 min, 1250 W) was introduced before fungal incubation with substrate.

Enzyme activity was measured according to a standard FPU method [18] and expressed as filter paper units (FPU) per mL of produced enzyme.

To evaluate fungal culture viability and overall quality, staining of liquid cultures was performed according to EloKITvita protocol [19], visualized with epifluorescence microscopy (Leica DM6000B, Leica Microsystems, Wetzlar, Germany) and analyzed with Image Pro Premier software (Mediacy, Rockville, MD, USA). For each sample a manual scanning at ×200 magnification was performed. At least 20 microscope fields of view were analyzed at ×2000 magnification.

### 2.5. Enzymatic Hydrolysis

Biomass hydrolysis was performed in 20 mL bottles containing 3% *w*/*v* of dry biomass (hay, wood or sawing residue chips, barley straw) or 9% *w*/*v* of wet biomass (algae) and 10 mL of 0.01 M sodium citrate buffer (pH ~5.5). The test was performed 3 times, each time having 2 technical repetitions. After mixing the biomass with the buffer, bottles were boiled for 5 min (1 atm) to reduce indigenous microorganisms and avoid lignification. After cooling, 0.1 mL of the produced enzyme was added to each bottle (0.2–0.3 FPU/mL final concentration). All bottles were incubated on an orbital shaker at 150 rpm at 30 °C for 24 h. Samples for sugar analyses were collected before enzyme addition and after incubation (24, 48 and 72 h for various biomass samples that were evaluated). All samples that were not processed immediately were stored in −18 °C.

### 2.6. Sugar Analysis

Sugar content after hydrolysis was measured using the dinitrosalicylic acid (DNS) method [20] and was expressed as total reducing sugar concentration. In brief, 0.1 mL of the liquid sample was mixed with 0.1 mL of 0.05 M sodium citrate buffer and 0.6 mL of 3,5-dinitrosalicylic acid and poured into glass tubes. Distilled water was used as the blank control. All samples were boiled for 5 min and cooled in cold water. Then, 4 mL of distilled water was added to each tube. Absorption was measured with a spectrophotometer at 540 nm (GENESYS 150, Thermo Fisher Scientific Inc., Waltham, MA, USA). To obtain absolute reducing sugar concentrations a standard curve of known sugar concentration was constructed. d-glucose standard solutions (Sigma-Aldrich, Darmstadt, Germany) were used as stock and quality control.

## 3. Results and Discussion

### 3.1. Fungal Cultivation Conditions in Biomass Substrate

As reported previously [14], one of the issues in efficient enzyme production and subsequent hydrolysis tests is the avoidance of indigenous microorganisms that can either compete with enzyme-producing fungi or simultaneously consume the released carbohydrates. To limit the growth of these microorganisms, sterility, including prior sterilization of fungal growth substrate, is a must. Reopening of the culture material and prolonged incubation can easily contaminate the extracts and cause erroneous results. Thus, to decrease the production costs of essential enzymes [7], short-term, aseptic cultivation is preferable.

**Figure 2:**
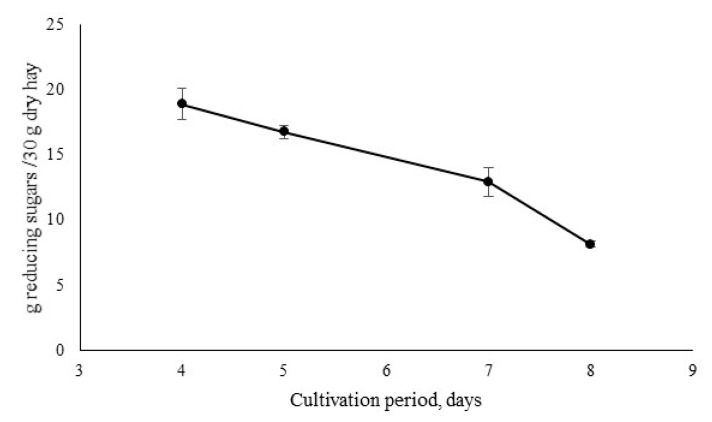
The amount of total reducing sugars produced after 24 h of hydrolysis from 30 g dry hay and 1 mL of enzyme cocktail that was obtained after 4, 5, 7 or 8 days of *I. lacteus* cultivation with hay biomass substrate as carbon source. Bars represent the average from 2 separate experimental repetitions.

**Figure 3:**
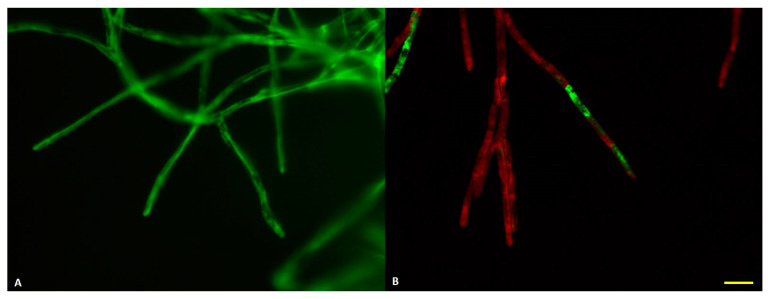
Viability staining of *Irpex lacteus* hyphae in 4 (**A**) and 8 (**B**) day old cultures. Green—metabolically active, red—inactive hyphae. Bar—10 µm.

To evaluate the impact of *I. lacteus* cultivation time on the quality of the extracted enzyme cocktail, proteins were precipitated and extracted from broth after 4, 5, 7 and 8 days of fungal incubation and tested for their quality to release carbohydrates from hay biomass. A shorter incubation period (1–3 days) was avoided since that is the time *I. lacteus* requires to develop extensive hyphae growth and no visible mycelia are formed (Figure S1). The results demonstrated that the ability of the produced lignocellulosic enzyme cocktails to release carbohydrates from cellulose and hemicellulose fractions decreased with the incubation time used for enzyme production (Figure 2), reaching only 43% of the efficiency after 8 days of cultivation when compared to a 4-day-old fungal protein extract. The enzyme cocktail that was obtained after 4 days of fungal growth on hay as a carbon source, in subsequent hay hydrolysis demonstrated 63% conversion rate into reducing sugars. These observations to some extent contradict with other studies where white-rot fungi produced the most active enzymes after 12 days of incubation [21]. The differences could be explained by previous assessment of only individual enzymes or their limited combinations [8,21] or the by more intense binding of the enzyme to carbohydrate source (hay) at later incubation stage and inefficient recovery of that enzyme fraction. In this study, the performance of the whole crude enzyme cocktail without purification and removal of any specific protein fraction was assessed. As previously reported [8], enzyme cocktails obtained from *I. lacteus* contain various enzymes. Thus, the apparent presence of certain factors at the initial biomass degradation stage seem to be essential. Moreover, qualitative epifluorescence microscopy analyses demonstrated that *I. lacteus* culture on day 4 demonstrated high metabolic activity with no inactive hyphae and a tendency to have high enzyme activity in hyphal tips (Figure 3A). At the same time, the 8-day-old culture had a visually higher amount of metabolically inactive hyphae with reduced activity in the tips (Figure 3B). As demonstrated earlier, most of the enzyme formation occurs at the tips of the fungi [22], thus, careful selection of cultivation time is of high importance in the setup of technology for lignocellulosic enzyme production. Furthermore, a general trend in the increase of culture pH was observed with increasing cultivation time (Table S1).

The highest enzyme activity and ability to perform hydrolysis was observed with enzyme cocktails that were obtained after fungal growth on hay substrate. Use of Avicel or wood residue chips produced lower quantity of protein (low or no precipitate after addition and incubation with ammonium sulphate) and did not result in more efficient enzymes even if the same substrate was used in enzymatic hydrolysis. A maximum of 0.14 g of fermentable sugar from hay biomass was obtained with the enzyme cocktail produced on Avicel substrate. Similar observations were made when wood chips were used as carbohydrate source for fungal growth and enzyme production. Moreover, longer incubation times were required to observe active hyphae formation on wood chips and Avicel (up to 8 days to develop mycelia in comparison to 4 days on hay), thus indicating that these substrates might be not suitable for enzyme production from fungi or need some additional pre-treatment. All further studies were performed only with hay as carbohydrate source for fungal growth and enzyme production.

### 3.2. Enzyme Extraction Efficiency

**Figure 4:**
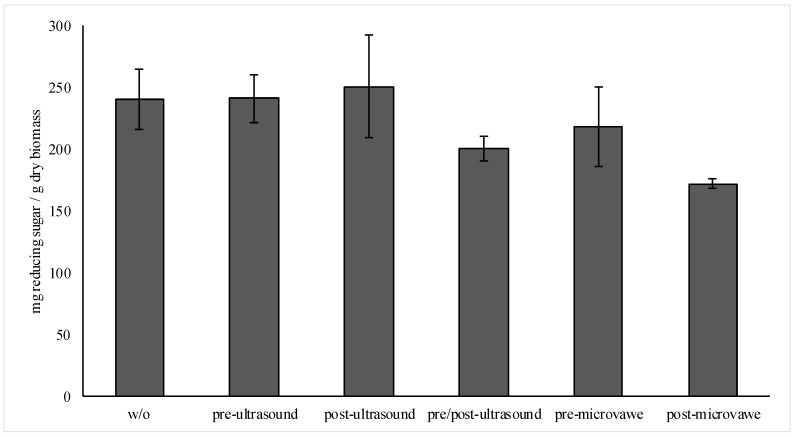
The amount of reducing sugar produced per g dry hay biomass when hydrolyzed with enzyme preparations that have been treated with ultrasound or microwave pre and post fungal cultivation and prior to enzyme precipitation and extraction. Bars represent the average of 6 to 12 individual tests; w/o—without any additional treatment step.

Mechanical, physical and chemical pre-treatment of biomass is essential for efficient hydrolysis [23]. Similarly, during the enzyme production, fungi need to access biomass and additional pre-treatment will aid in opening of the lignin–carbohydrate complex to favor more rapid enzyme formation. At the same time, many of the proposed treatments are incompatible with fungal growth and enzyme production technologies, especially when the sterility, neutralization strategies, removal of chemicals and price-to-value issues need to be considered. From non-reagent-based approaches, microwave irradiation has been demonstrated to be efficient in increasing enzyme access to cellulose [24] and ultrasound for rapture of hydrogen bonds [23]. The results of this study (Figure 4) showed that microwave treatment does not possess any significant impact when used with prior fungal inoculation. The produced enzyme cocktail released 0.22 ± 0.03 g sugar/g dry biomass while the control extract without any pre-treatment reached 0.24 ± 0.02 g/g dry biomass. Microwave treatment after cultivation of fungi (to yield enzyme detachment from biomass) produced only 0.17 ± 0.004 g fermentable sugar per g of dry biomass in subsequent hydrolysis tests with hay, meaning that a lower quality enzyme cocktail was produced and could be linked with partial degradation of the proteins. In the meantime, the introduction of ultrasound increased the amount of fermentable sugars to be released with the respective enzyme cocktail, yielding 0.25 ± 0.04 g/g dry biomass when ultrasound after incubation of fungi was used. Additional cellulase activity measurements for the enzyme cocktails without post-ultrasound treatment yielded on average 16.7 FPU/mL while enzymes that had post-ultrasound treatment yielded 29.8 FPU/mL. Thus, this indicated a possibly more efficient detachment of enzymes from biomass.

Further assessment of ultrasound treatment time (2 min vs. 4 min) did not demonstrate any increase in enzyme efficiency at 30 Hz (0.22 g fermentable sugar/g dry biomass) and even had a decrease in efficiency from 3 to 14% when the intensity was increased from 30 Hz to 50 Hz.

Even if no significant difference in fermentable sugar yields was observed in pre- and post- treatments with ultrasound (*p* > 0.05), post-treatment was still favored, since it 1) had less impact on sterility, and 2) could favor situations when fungi formed solid structures on biomass substrates during growth (Figure S1).

### 3.3. Production of Fermentable Sugars

Efficient release of fermentable carbohydrates from lignocellulosic biomass is a complex process of multiple enzymes targeting various bonds in the lignocellulosic material and additional proteins supporting the work of the degrading enzymes [25]. Thus, the efficiency of the produced enzyme cocktails should be addressed by their ability to form the overall product and not by an activity of an individual enzyme per se. To evaluate the fermentable carbohydrate release potential in hydrolysis with the produced enzyme cocktail (Figure 4, post-ultrasound), biomass substrates with various recalcitrance levels were tested. These included green freshwater microalgae, wood residue chips, sawing residue chips and barley straw (Figure 1). All were selected due to being representative renewable resources suggested by EU RED [1] or being abundant in the environment with high regeneration potential. Enzymatic hydrolysis with novel enzyme cocktails was combined with mild pre-treatment (mechanical size reduction and heat treatment at 1 atm) [26] to support the concept of simple, low-energy-consuming and scalable technology. The highest conversion yields were obtained from hay substrate where more than 20% of biomass dry weight was converted to fermentable sugar within 24 h of incubation (Table 1). Only a slight increase (3.7% more after 48 h and 6.8% after 72 h) was observed with prolonged incubation time, thus indicating that hay substrate is easily accessible for the enzymes and 24 h hydrolysis is sufficient.

Barley straw and microalgae substrates also demonstrated acceptable conversion yields, yielding more than 0.1 g fermentable sugar from each g of dry substrate. However, apparent importance of hydrolysis time was observed for these substrates, since 72 h hydrolysis increased total fermentable carbohydrate yields for 21.3% (for algae) and 37.1% (for straw) when compared to 24 h hydrolysis.

The lowest recoveries were obtained for wood and sawing residue chips, yielding less than 0.06 g fermentable sugar per g dry biomass. Again, a certain increase (18.6% and 34.4% for wood and sawing chips respectively) in produced sugar yields was observed with prolonged hydrolysis. Apparent lower recoveries and longer required incubation times indicated the need for a more intense pre-treatment technique to aid fungal access to cellulose and reduce the potential impact of substrate structural factors causing recalcitrance, e.g., cellulose surface area, cellulose crystallinity, pore size and volume [27]. Furthermore, lack of mannan-degrading enzymes in the cocktail might reduce the efficiency due to the potential presence of softwood in the wood and sawing residue [28].

Within this study we demonstrated that enzyme cocktails produced from *I. lacteus* only after 4 days of incubation can be highly effective and with an additional prior ultrasound treatment, precipitation and extraction yield more than 0.2 g of fermentable sugar per g dry biomass. At the same time, enzyme complexes have to be combined with suitable pre-treatment if various biomass substrates are used.

Natural enzyme cocktails contain a wide range of proteins which can be complicated to characterize and produce at constant quantities when such products are manufactured at an industrial level. At the same time, we have demonstrated that careful and detailed control and maintenance of fungal incubation and extraction conditions with the regular evaluation of overall performance can be an effective tool to produce lignocellulose-degrading enzymes industrially or, better, at the sites of saccharification, thus minimizing the impact of commercial products.

## 4. Conclusions

Efficient fermentable carbohydrate-releasing enzyme cocktails from the white-rot basidiomycete *Irpex lacteus* can be produced in only 4 days of fungal cultivation with hay as substrate. The activity of the produced enzyme cocktails decreased with the cultivation time of *I. lacteus*.

The obtained enzyme cocktails had higher efficiency when low intensity post-cultivation ultrasound was used to release the enzymes into the liquid prior to subsequent precipitation. Microwave treatment had no positive impact on enzyme product efficiency to release carbohydrates from biomass.

Enzymatic hydrolysis with produced enzyme cocktails yielded more than 0.2 g of fermentable sugar per g hay in 24 h and 0.22 g in 72 h. More than 0.11 g of fermentable sugar was obtained in 72 h hydrolysis with straw and microalgae substrates.

The results demonstrated that the production of enzymes is more sensitive than previously described, e.g., in terms of fungal growth, potential pollution and incubation conditions. Nevertheless, under certain standardization, effective natural enzyme cocktails can be in situ produced for biomass enzymatic hydrolysis.

## Figures and Tables

**Table 1:** Milligrams of fermentable sugar released per g dry biomass with enzyme cocktail obtained from *I. lacteus* with introduced post-cultivation ultrasound treatment.

Substrate	Incubation Time		
	24 h	48 h	72 h
Hay	207.5 ± 7.2	215.3 ± 4.8	221.7 ± 8.0
Wood residue chips	50.3 ± 4.3	52.4 ± 4.2	59.6 ± 6.2
Sawing residue chips	24.8 ± 0.7	28.0 ± 1.2	33.4 ± 1.8
Barley straw	81.9 ± 6.6	104.3 ± 7.9	112.3 ± 10.3
Green algae (dried)	93.7 ± 13.2	110.2 ± 5.7	113.6 ± 3.9

Standard deviation represents the average value from three independent tests each having 2 repetitions.

## Supplementary Materials

The following are available online at https://www.mdpi.com/2309-608X/6/4/256/s1, Figure S1: Fungal growth in flasks, Table S1: Changes in efficacy of enzymes.
