# Supplementary material for: Bioprospecting White-Rot Basidiomycete Irpex lacteus for Improved Extraction of Lignocellulose-Degrading Enzymes and Their Further Application
Source: J Fungi (Basel). 2020 Oct 29;6(4):256. doi: 10.3390/jof6040256 (PMC7712641; doi:10.3390/jof6040256)
Supplement: Supplementary file 1 [file jof-06-00256-s001.pdf]

Journal of Fungi

MDPI

# Supplementary Materials: Bioprospecting White-Rot Basidiomycete *Irpex lacteus* for Improved Extraction of Lignocellulose-Degrading Enzymes and Their Further Application

**Table S1.** Changes in efficacy of enzymes (ability to release fermentable sugars from hay biomass) and enzyme-producing culture pH with respect to enzyme-producing culture incubation time.

| Time, Days | g Fermentable Sugar from mL Enzyme | Average pH in Cultures |
|---|---|---|
| 4 | 18.9 ± 1.2 | 6.8 |
| 5 | 16.7 ± 0.5 | 7.3 |
| 7 | 12.9 ± 1.1 | 6.9 |
| 8 | 8.1 ± 0.3 | 7.2 |

The data represent the average value from 3 independent tests.

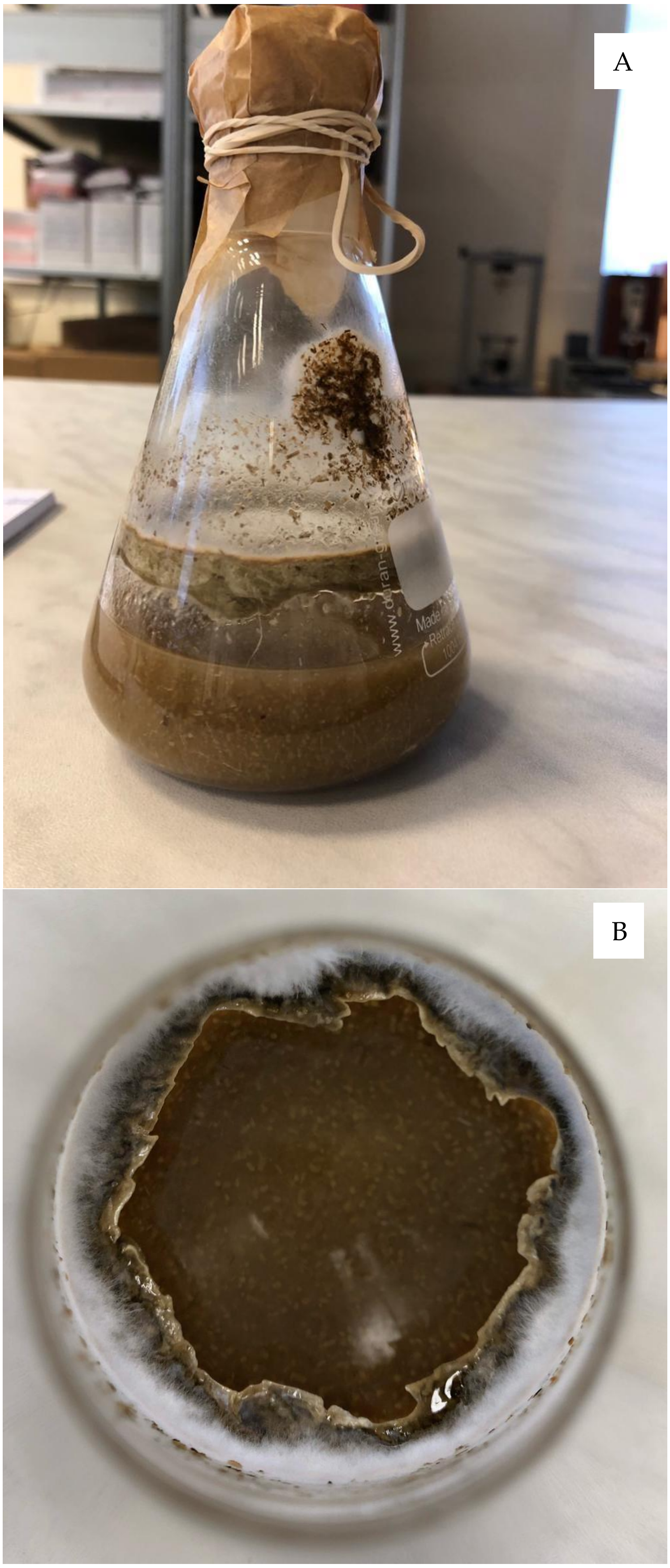


**Figure S1.** Fungal growth in the flasks with biomass substrate and formation of growth zone at the top (**A**) or in the bottom (**B**) part of the flask depending on the incubation time. Longer incubation (bottom) resulted in more intense fungal biomass and less effective enzyme cocktails.
